# Structural basis for the selective inhibition of the PI3KC3-C2 complex by Rubicon in endolysosome maturation and mitophagy

**DOI:** 10.64898/2026.08.07.743589

**Published:** 2026-08-10

**Authors:** Minghao Chen, Aniketh Bishnu, Yongjia Duan, Julia F. Riley, Qingyang Ni, Aaron Joiner, Isaac J. Allen, Erika L. F. Holzbaur, Ian G. Ganley, James H. Hurley

## Abstract

Rubicon is a negative regulator of autophagy and the endolysosomal network (ELN) and an antagonist of the class III phosphatidylinositol 3-kinase complex II (PI3KC3-C2). Inhibition of Rubicon is considered a potential means to therapeutically upregulate autophagy and the ELN to treat Parkinson’s disease and other conditions characterized by autophagic and ELN dysfunction. Rubicon is specific for the UVRAG-containing PI3KC3-C2 over the purely autophagic ATG14- containing PI3KC3-C1 complex. Here, we determined the high-resolution cryo-electron microscopy structure of PI3KC3-C2 in complex with the PI3KC3-binding domain (PIKBD) of Rubicon and compared it to cryo-EM structures of unbound PI3KC3-C2 and PI3KC3-C1. Rubicon binds directly to PI3KC3-C2 only via the BARA domain of the BECN1 subunit, which is common to both C1 and C2. The selectivity of Rubicon for the PI3KC3-C2 complex over the PI3KC3-C1 complex is attributed to a conformation of the BECN1 ^BARA^ domain induced by UVRAG, rather than to direct contact with UVRAG or direct antagonism by the ATG14 subunit of PI3KC3-C1. Targeted disruption of the Rubicon:PI3K3-C2 structural interface by site-directed mutations enhances mitophagic activity in human epithelial cells to levels comparable to those observed in Rubicon knockout (KO) cells. Similarly, disruption of the interaction in Rubicon-overexpressing hippocampal neurons restored lysosomal flux to wild-type levels. These data show that suppressing the function of PI3K3- C2 can fully account for the negative regulatory effects of Rubicon in the autophagy and ELN pathways.

**Significance Statement:**

Endolysosome maturation and autophagosome-lysosome fusion require the production of phosphatidylinositol 3-phosphate (PI(3)P) by the class III phosphatidylinositol 3-kinase complex II (PI3KC3-C2). Rubicon is a key negative regulator of endolysosomes and autophagy that suppresses PI3KC3-C2 activity. Here, we reveal in atomistic detail how Rubicon selectively recognizes PI3KC3-C2. Disrupting the Rubicon–PI3KC3-C2 interaction restores mitophagy and enhances lysosomal activity to the same extent as Rubicon gene deletion, establishing that PI3KC3-C2 inhibition fully accounts for the biological regulatory effects of Rubicon in the autophagy and lysosome pathways.

## Full Text Availability

The license terms selected by the author(s) for this preprint version do not permit archiving in PMC. The full text is available from the preprint server.

